# Minimally invasive microbiopsies: a novel sampling method for identifying asymptomatic, potentially infectious carriers of *Leishmania donovani*

**DOI:** 10.1016/j.ijpara.2017.02.005

**Published:** 2017-09

**Authors:** Oscar David Kirstein, Ibrahim Abbasi, Ben Zion Horwitz, Laura Skrip, Asrat Hailu, Charles Jaffe, Lynlee L. Li, Tarl W. Prow, Alon Warburg

**Affiliations:** a Department of Microbiology and Molecular Genetics, The Institute for Medical Research Israel-Canada (IMRIC), The Kuvin Centre for the Study of Infectious and Tropical Diseases, The Hebrew University – Hadassah Medical School, The Hebrew University of Jerusalem, 91120, Israel; b Department of Biostatistics, School of Public Health, Yale University, 60 College Street, New Haven, CT 06520, USA; c Department of Microbiology, Immunology & Parasitology, Faculty of Medicine, Addis Ababa University, P.O. Box 9086, Addis Ababa, Ethiopia; d Dermatology Research Centre, The University of Queensland School of Medicine, Translational Research Institute, Brisbane, QLD 4012, Australia

**Keywords:** Asymptomatic carriers, *Leishmania donovani*, Microbiopsy, Phlebotomine sand flies, Visceral leishmaniasis, Xenodiagnosis

## Abstract

Visceral leishmaniasis (VL) is a potentially lethal, sand fly-borne disease caused by protozoan parasites belonging to the *Leishmania donovani* species complex. There are several adequate methods for diagnosing VL, but the majority of infected individuals remain asymptomatic, comprising potential parasite reservoirs for transmission of the disease. The gold standard for assessing host infectiousness to biting vector insects is xenodiagnosis (i.e. scoring infection rates among insectary-reared insects that had fed on humans suspected of being infected). However, when it comes to sand flies and leishmaniasis, xenodiagnosis is an intricate operation burdened by logistical hurdles and ethical concerns that prevent its effective application for mass screening of widely dispersed communities, particularly in rural regions of underdeveloped countries. Minimally invasive microbiopsy (MB) devices were designed to penetrate the skin to a depth of ∼200 µm and absorb blood as well as skin cell lysates, mimicking the mode by which phlebotomine sand flies acquire blood meals, as well as their composition. MBs taken from 137 of 262 volunteers, living in endemic VL foci in Ethiopia, detected *Leishmania* parasites that could potentially be imbibed by feeding sand flies. Although the volume of MBs was 10-fold smaller than finger-prick blood samples, *Leishmania* DNA detection rates from MBs were significantly higher, implying that skin, more often than blood, was the source of parasites. Volunteers with histories of VL were almost as likely as healthy volunteers to test positive by MBs (southern Ethiopian focus: 95% CI: 0.35–2.59, *P* = 1.0. northern Ethiopian focus 0.87: 95% CI: 0.22–3.76, *P* = 1), suggesting the importance of asymptomatic patients as reservoirs of *L. donovani*. Minimally invasive, painless MBs should be considered for reliably and efficiently evaluating both *L. donovani* infection rates among large numbers of asymptomatic carriers and their infectiousness to blood-feeding sand flies.

## Highlights

• Microbiopsy devices were designed to assess the infectiousness of asymptomatic *Leishmania donovani* carriers.
• The microbiopsy devices sample both skin tissues and blood, as do pool-feeding phlebotomine sand flies.
• Devices were tested on human volunteers in Ethiopia and proven effective, surpassing the sensitivity of finger-pricks.

## Graphical abstract

**Figure:**
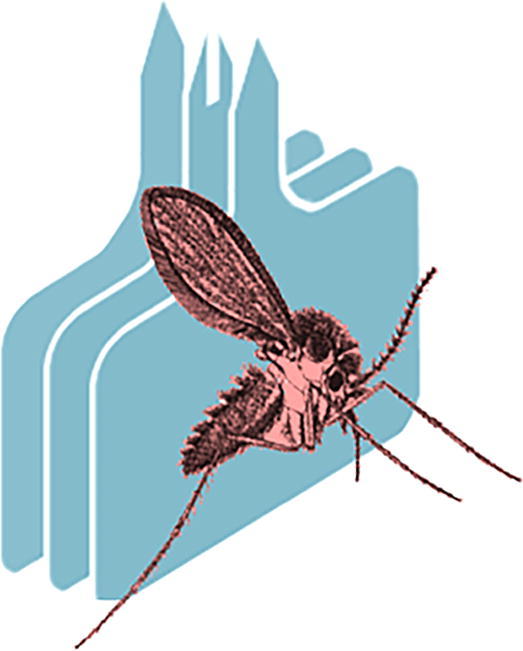

## 1 Introduction

Visceral leishmaniasis (VL) is caused by disseminated infections with eukaryotic *Leishmania donovani* (complex) parasites. An estimated 390,000 VL cases occur annually, over 90% of which are concentrated in the Indian sub-continent, eastern Africa and Brazil (Alvar et al., 2012). Distinct modes of transmission characterize the two causative parasite species responsible for VL. *Leishmania donovani infantum* in Latin America, Europe, the Middle East and North Africa is transmitted zoonotically with dogs serving as the principal reservoir while *Leishmania donovani donovani* in the Indian subcontinent and eastern Africa is transmitted anthroponotically between humans (Chappuis et al., 2007). The worst affected African countries are Sudan and Ethiopia (Alvar et al., 2012, Gadisa et al., 2015). To understand the anthroponotic transmission dynamics of *L. donovani*, it is crucial to diagnose not only the VL cases that normally comprise a small minority of the infected population, but also to identify the asymptomatic carriers with high parasitemias accessible and, therefore, potentially infectious to biting sand flies (Miller et al., 2014, Singh et al., 2014, Hirve et al., 2016).

Several serological assays for VL have been developed but none serve as “stand-alone” tests (Boelaert et al., 2008). Serological diagnosis is achieved using Freeze Dried – Direct Agglutination Tests (FD-DAT) or rK39 strip tests. Parasitological confirmation of VL is achieved by microscopic examination of Giemsa-stained splenic aspirate smears (96% sensitivity) (Boelaert et al., 2008, ter Horst et al., 2009). Targets for PCR-based diagnosis of *Leishmania* infections in humans include kinetoplast DNA (kDNA) minicircles (Abbasi et al., 2013), the ssrRNA gene (van Eys et al., 1992), the internal transcribed spacer 1 (ITS1) (el Tai et al., 2000) and the spliced leader sequence (van Eys et al., 1992). Further analysis of the PCR amplicon is required for species identification (e.g. restriction cut analysis of ITS1 (el Tai et al., 2000), high resolution melt analysis of kDNA and 7SL (Bensoussan et al., 2006) or DNA sequencing (el Tai et al., 2000, Bensoussan et al., 2006, Abbasi et al., 2013).

Some blood-feeding insects (e.g. triatomine bugs, vectors of Chagas disease) obtain blood by direct cannulation of blood vessels (vessel feeders). On the other hand, the mouthparts of the phlebotomine vectors of leishmaniasis are relatively short (250–350 µm) allowing penetration no deeper than the dermis (100–150 µm), which is relatively poor in blood vessels (Lewis, 1987, Brinson et al., 1993, Bates, 2007). Sand flies induce minute superficial hemorrhages by macerating skin cells and cutting dermal capillaries with their serrated mouthparts. They imbibe blood that drains into the resultant hematomas together with skin cell lysates (pool-feeding or telmophagous). *Leishmania* parasites are not blood-borne parasites per se, therefore pool-feeding makes sand flies particularly likely to ingest *Leishmania* amastigotes inhabiting resident dermal macrophages, despite the small volume of their blood meals (e.g. 0.59 µl on average for *Phlebotomus orientalis*, a vector of VL in Sudan and Ethiopia) (Seblova et al., 2013).

Host infectiousness is optimally assessed based on the infection rates of insectary-reared vectors that had fed on it (xenodiagnosis). Unfortunately, xenodiagnosis for leishmaniasis is an intricate operation encumbered by technical, logistical and ethical hurdles that preclude its mass application for screening large communities in remote localities (Hirve et al., 2016). Here we describe a novel approach for detecting *L. donovani* infections and determining the potential of humans to infect biting sand flies. We used absorbent microbiopsy (MB) devices that painlessly collect minute skin/blood samples similar in composition to sand fly blood meals. These MB devices were developed by two of the authors (T. Prow and L. Li), utilizing state-of-the-art computer modeling, feeding into subtractive and additive manufacturing techniques to generate a simple, albeit sophisticated tool for use under field conditions (Lin et al., 2013). We suggest that absorbent MBs can serve as surrogates for sand flies in the xenodiagnosis of VL. Similar MBs can potentially serve for the xenodiagnosis of other vector-borne diseases caused by skin-dwelling parasites such as onchocerciasis (river blindness).

## 2 Materials and methods

### 2.1 Study sites

The field studies were conducted in two relatively well-studied VL foci in Ethiopia where clinical, epidemiological and entomological studies have been conducted in the past (Gebre-Michael and Lane, 1996, Hailu et al., 2009, Abbasi et al., 2013). The first study site was Aba Roba (5°18′42.53″N/37°24′31.12″E), Konso district, southwestern Ethiopia. The incriminated vectors of *L. donovani* in Konso are *Phlebotomus martini* and *Phlebotomus celiae* (Gebre-Michael and Lane, 1996). The second study area was the villages around the town of Sheraro (14°23′41″N/37°46′15″E), Tahtay Adiyabo district, Tigray region, northern Ethiopia where the vector species of *L. donovani* is *Ph. orientalis* (Gebresilassie et al., 2015).

### 2.2 Ethical considerations

Informed consent was sought from all the adults recruited for the study. Consent for inclusion of young children was obtained from parents or guardians. Study procedures were approved by the ethical review committees of the Medical Faculty, Addis Ababa University, Ethiopia and the National Research Ethics Review Committee (NRERC) at the Ethiopian Ministry of Science and Technology.

### 2.3 MB devices

**Fig. 1:**
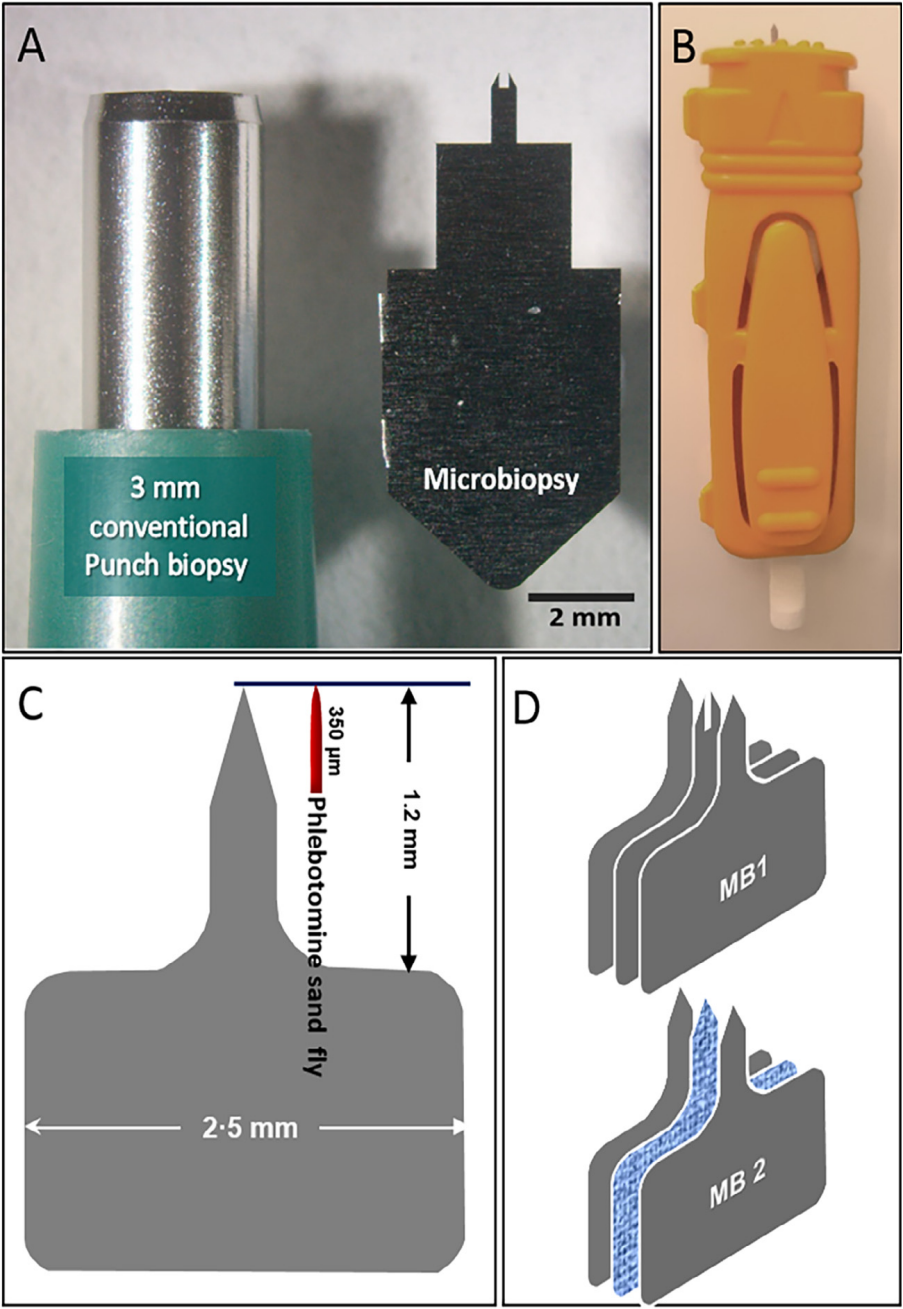
Design and structure of the microbiopsy (MB) devices. (A) Size comparison between a conventional 3 mm skin punch and a MB device. (B) Assemblage of the MB devices into a spring-loaded applicator. (C) Size comparison between the MB device and the mouthparts of sand flies. (D) Assembly of the MB devices. MB device type 1 (MB1) has a bifurcated central plate for sampling (mostly) skin. MB type 2 (MB2) has an absorbent central layer made of either polyethersulfone (PES) or Whatman paper No. 1. MB1s were produced by laser cutting to obtain precise sizes. Photo etching was used to produce MB2s with razor-sharp cutting edges.

We evaluated two prototypes of MB devices made of 50 µm medical grade stainless steel plates (Fig. 1). The first type, MB1 (Fig.1D), comprised two pointed outer plates and a central bifurcated one (Lin et al., 2013). The second type, MB2 (absorbent, Fig.1D), consisted of two external plates produced by photo-etching, with a central absorbent layer made of either Polyethersulfone (PES) membrane (Supor®, Sigma–Aldrich, Australia) or Whatman (USA) paper No. 1. The cutting bits were fitted into a disposable spring-loaded plunger mechanism for accurate deployment (Fig.1B). The MB devices were sealed in Stericlin® Tyvek® 1073 B pouches (Med-Con Pty. Ltd, Australia) and sterilized with gamma irradiation. MB samples were extracted from the arm, the nape of the neck and the cheek.

Prior to extraction of MB samples, the skin was wiped with 70% ethanol. The spring mechanism was compressed, the device was pressed against the skin, the plunger was released and the device held firmly in place for approximately 30 s to assure efficient absorption of blood and skin cell lysates.

### 2.4 Capillary blood samples

Blood samples were obtained by finger pricks (FPs) using disposable blood lancets and blotted on Whatman 3MM filter papers that were kept dry at room temperature.

### 2.5 DNA extraction

The cutting plates of MB devices were removed from the applicator immediately after use, placed in 1.5 ml micro-centrifuge tubes containing 200 μl of DNA extraction buffer and stored at room temperature.

Two paper-punch disks (*r* = 3 mm, calculated to have been saturated with approximately 20 µl of FP blood each) of Whatman No. 1 filter papers were placed in 1.5 ml micro-centrifuge tubes with 200 µl of DNA extraction buffer. Subsequent DNA extraction and purification were performed as described previously (Abbasi et al., 2013). DNA concentrations were measured using a full spectrum micro-volume UV/Vis spectrophotometer (NanoDrop 2000c, Thermo Scientific, Surrey, UK).

### 2.6 Quantitative real-time kinetoplast DNA PCR (qRT-kDNA PCR)

Real-Time hot-start PCR was performed with an Absolute Blue qPCR kit (Thermo Scientific) based on SYBR green detection using a real-time PCR thermocycler (Rotor-Gene 6000, Qiagen, Hilden, Germany). The qRT-PCR mixture contained 10 µl of the 2x concentrated absolute blue solution with 1 µM each of *Leishmania* kDNA minicircle-specific primers JW11 and JW12, and template DNA (2 µl) (total volume of 20 µl). The PCR protocol, calibration curves and analysis of the results were as previously described (Abbasi et al., 2013). To verify adequate extraction of DNA and the absence of putative PCR inhibitors, qRT-PCR of the human mitochondrial cytochrome b gene (*cyt b*) was performed (Abbasi et al., 2009).

### 2.7 Reverse transcription PCR

Total RNA was extracted from a number of MBs using the TRI Reagent® Protocol (Sigma–Aldrich). The cutting bit of the microbiopsy device containing blood/skin tissues was incubated in 0·5 ml of Tri-Reagent solution for 15 min, followed by purification using chloroform and RNA precipitation from the aqueous layer using isopropanol. The precipitated RNA was washed with 70% ethanol and resuspended in RNAase-free double distilled water. The RNA served as a template for reverse transcription using a RevertAid First Strand cDNA Synthesis Kit (Thermo Fisher Scientific, MA, USA). Approximately 1 ng of total RNA was incubated with reverse transcriptase in the presence of oligo (dT)_18_ primers. Two negative controls were included: (i) without reverse transcriptase, and (ii) without RNA template.

### 2.8 Keratin and β-actin gene products PCR amplification

PCR amplification of a 105 bp DNA segment from the first strand cDNA of the skin-specific keratin gene product was performed using the direct primer *Keratin14D* (CCTCCTCCCAGTTCTCCT) and reverse primer *Keratin14R* (ACACCACCTTGCCATCG). Keratin 14 (K14) is an intermediate filament that is expressed in squamous epithelia. This gene is exclusively expressed in stratified epithelium and its promotor is commonly used to drive transgene expression in skin. The skin-specific expression of K14 is conferred by the promoter segment (Staggers et al., 1995). To verify the ample presence of first strand cDNA, an 800 bp segment of the human β-actin housekeeping gene was also amplified using the forward primer: ATCTGGCACACCTTCTACAATGA and the reverse primer: CGTCATACTCCTGCTTGCTGATCCAC) (Lin et al., 2013). The presence of blood was readily verified visually in all MB2s.

### 2.9 Data analysis

Data obtained from the two studies were analyzed separately. The correlation between parasitemia levels measured by MBs from different anatomical sites (i.e. the face and neck) was analyzed using Pearson's correlation coefficient. For additional analyses, an individual was considered positive for *Leishmania* parasites by MB if any MB sample (face or neck) was PCR-positive. Unadjusted odds ratios were calculated to determine the likelihood of a positive MB result among recovered VL cases relative to healthy individuals. Infection intensity (parasitemia), as measured by qRT-kDNA PCR, was categorized as 0 (uninfected), 1–10, 11–100, and >100 parasites per ml. Chi-square or Fisher’s Exact Tests (FET) were used to assess agreement in infection diagnoses between MB and FP and to compare the distributions of infection intensities detected by the two methods. All analyses were performed using R version 3.2.1 (R Foundation for Statistical Computing, Vienna, Austria), IBM SPSS statistics, version 23 for Windows and Microsoft® Office Excel 2013. Analyses with P values ≤0.05 were considered significant. The Raw data is included in Supplementary Table S1.

## 3 Results

### 3.1 MBs comprise skin and blood tissues

**Fig. 2:**
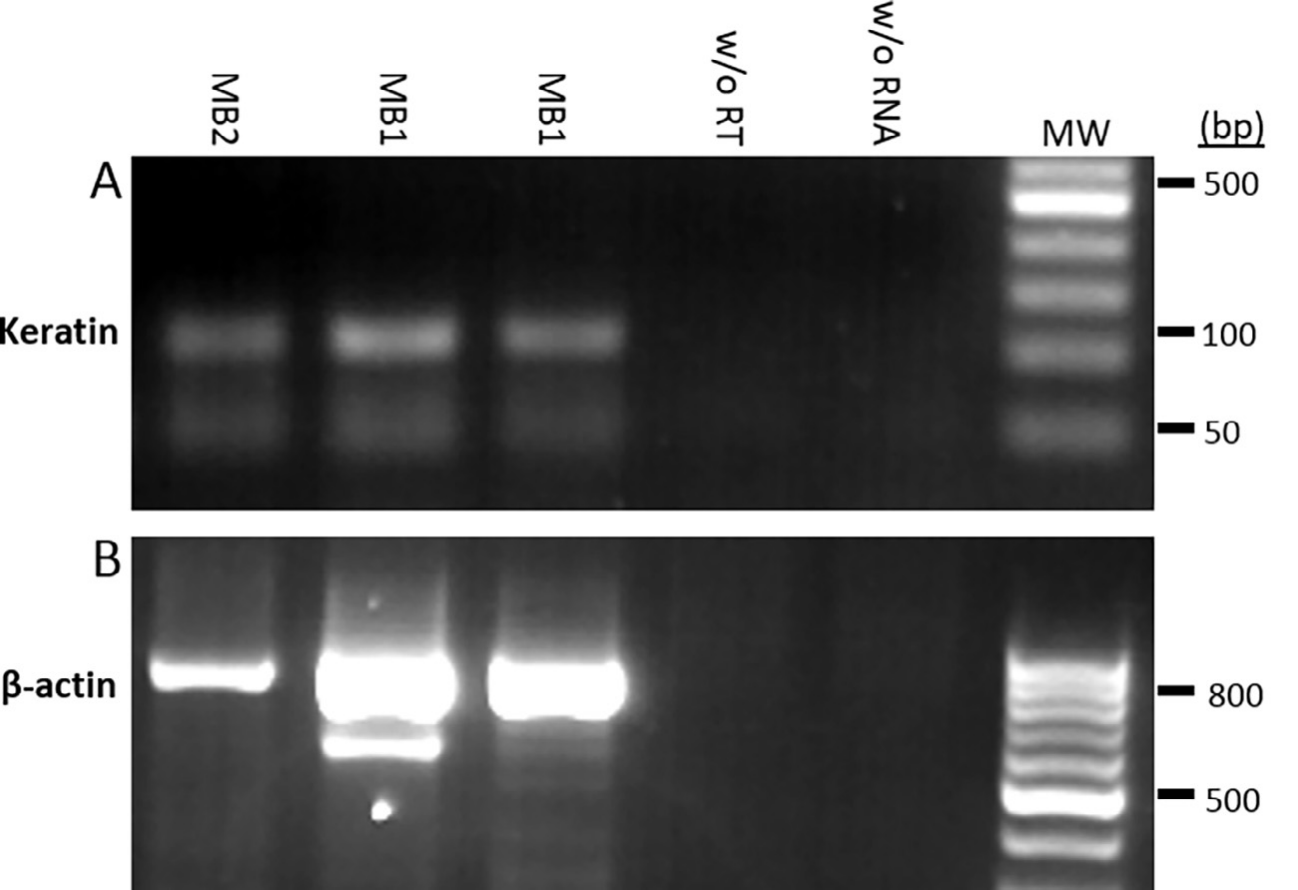
Reverse transcriptase PCR of mRNA extracted from microbiopsies (MBs) for detection of skin cells. The first strand cDNA was prepared from total mRNA yields of: Lane 1, MB type 2 (MB2); Lanes 2,3, MB type 1 (MB1); Lane 4, negative control without (w/o) reverse transcriptase (RT); Lane 5, negative control without mRNA; MW, DNA size markers. (A) Keratin (skin-specific) cDNA, (107 bp). (B) β–Actin (housekeeping gene) cDNA (800 bp).

To confirm that MBs extract both blood and skin tissue, we performed *K14* (105 bp) -specific reverse transcription-PCR (Fig.2A). The human β-actin housekeeping gene was also reverse transcribed showing as a well-defined 800 bp band on agarose gels (Fig.2B). Results confirmed that both MB1 and MB2 devices extract skin tissues. The presence of blood was confirmed by eye.

### 3.2 Leishmaniasis patients

**Fig. 3:**
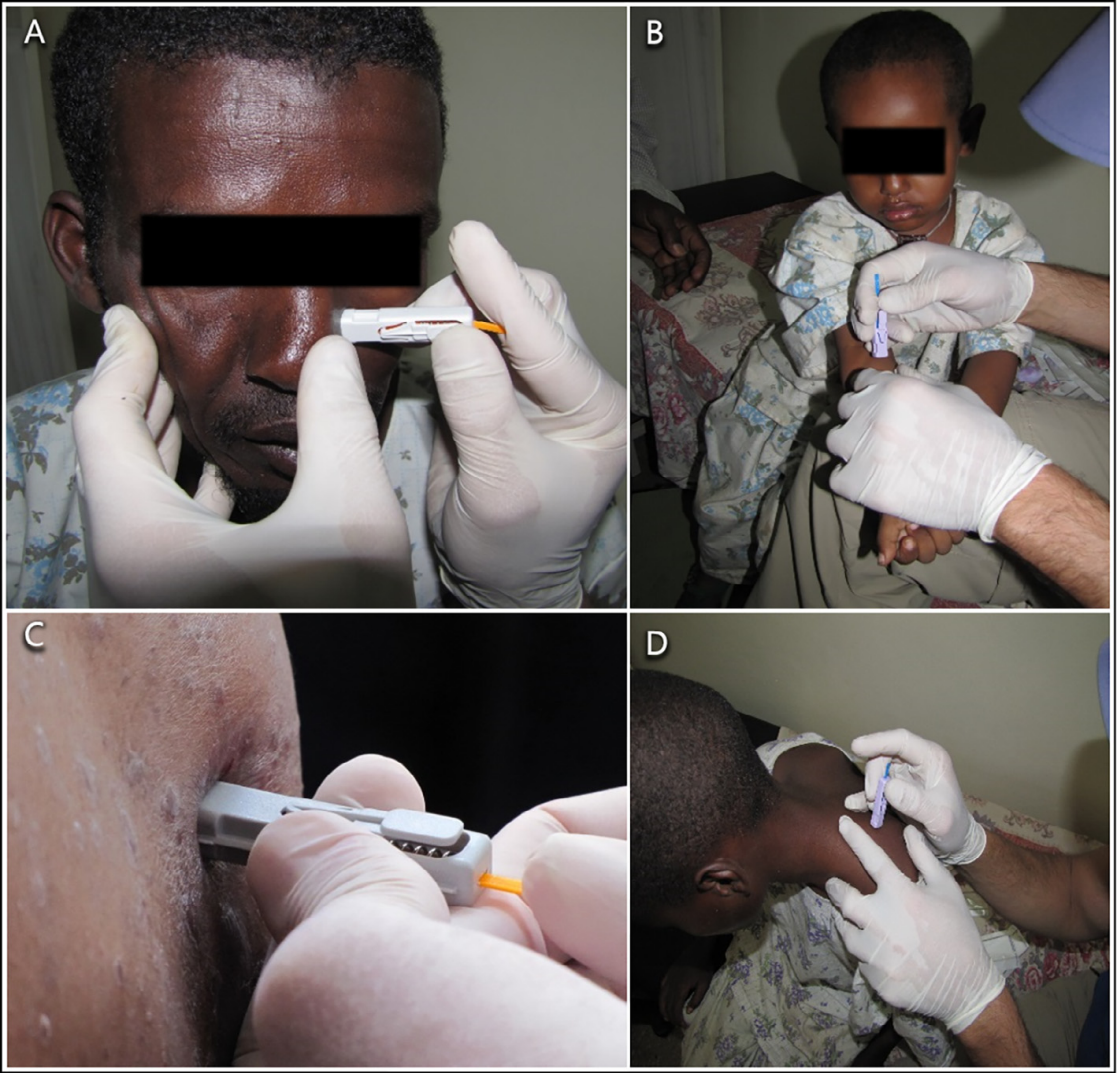
Photos taken during a study on visceral leishmaniasis (VL) patients in Ethiopia in 2015. (A) Microbiopsy type 2 (MB2) taken from the nose. (B) MB2 from the arm of a 5 year old child diagnosed with VL. (C) Close-up of a MB2 taken from a cutaneous leishmaniasis (CL) lesion caused by *Leishmania aethiopica* (species identified by DNA sequencing of the *Leishmania* internal transcribed spacer 1 (ITS1) gene.) (D) Microbiopsy sample being taken from the nape of the neck of a child VL patient.

Sampling of leishmaniasis patients was performed at the Black Lion Hospital in Addis Ababa, Ethiopia and the Leishmaniasis Research & Treatment Centre in Arba Minch (southern Ethiopia). Ten consenting patients were sampled; seven of them were diagnosed less than 24 h prior to sampling and had not been treated (pre-treatment); one patient was diagnosed with post-kala azar dermal leishmaniasis (PKDL), a chronic form of cutaneous leishmaniasis (CL) that develops after treatment and resolution of VL; one patient was co-infected with the human immunodeficiency virus (HIV) and *L. donovani*; and one was a case of CL caused by *Leishmania aethiopica* (Table 1). MB samples were obtained from the face, the nape of the neck and the forearm using MB1 and MB2 devices (Figs. 1D, 3). FP blood was collected to monitor “pure” blood parasitemias assuming the probable lack of skin parasitemias in the fingertips. Results of human *cyt b* PCRs were positive for all samples. Total DNA concentrations averaged 4.64 (S.D. = 4.11) ng/µl for MB1 s and 57.60 (S.D. = 8.98) ng/µl for MB2s. The coefficient of variation (CV) was CV = 0.1 for MB2 versus CV = 1.3 for MB1.

**Table 1:** Results of quantitative real time kinetoplast DNA (qRT-kDNA) PCR targeting *Leishmania* DNA extracted from samples collected with microbiopsy (MB) device types 1 and 2 (MB1, MB2) and finger pricks (FP) from 10 hospitalized leishmaniasis patients during the pilot study.

No^a^	Parasite concentrations (per ml)						Patient information
	MB type	MB Location				FP	
		Arm	Back	Nape of Neck	Face		
							
1	MB1	0	10 (0.1)	–	–	278	Pre-treated VL+
	MB2	60	30 (34·5)	–	–		
2	MB1	80	–	10	–	92,714	Pre-treated VL+
	MB2	3,920	–	2,850	–		
3	MB1	10	–	10	–	2,810	Pre-treated VL+
	MB2	2,710	–	80	–		
4	MB1	10	–	–	–	279	Pre-treated VL+
	MB2	70	–	–	–		
5	MB1	20	–	0	0	405	Pre-treated VL+
	MB2	20	–	260	930		
7	MB1	–	–	–	10	829	Pre-treated VL+
	MB2	20	–	–	340		
6	MB1	20	–	–	0	18	VL+ 6th day of treatment with Sodium stibogluconate
	MB2	70	–	–	10		
8	MB1^b^	20,130	–	–	–	>10^6^	Co-infection VL+ HIV, hard skin nodules, signs of PKDL
	MB2	1,080	–	–	–		
9	MB1^c^	–	–	–	1,160	7,156	PKDL Grade 1
	MB2	–	–	–	650		
10	MB1	–	60	–	–	250	Diffuse CL, 3 years treatment
	MB2	–	10	–	–		

VL, visceral leishmaniasis; HIV, human immunodeficiency virus; CL, cutaneous leishmaniasis.

a Patients 1 to 8 were diagnosed serologically using rK39 rapid tests followed by splenic punctures.

b MB1 device was applied to a papular post-kala azar dermal leishmaniasis (PKDL) lesion (scattered lesions were observed only on the face restricted to areas around the nose and mouth).

c MB1 device was applied directly on the surface of a hard skin nodule in the forearm.

The qRT-kDNA PCR results consistently demonstrated *Leishmania* DNA using the different sampling methods except for three MB1 samples (indicating the possible absence of parasites at the locations probed; Table 1). MBs taken from the borders of skin lesions caused by *L. aethiopica* also detected *Leishmania* DNA (Table 1). Capillary blood parasitemias detected by FPs were generally higher than those detected by MBs. ITS1 PCR products from three MB samples, two of which were extracted from VL patients and one from a CL patient, were sequenced, confirming the causative agents were *L. donovani* and *L. aethiopica,* respectively (data not shown).

### 3.3 Asymptomatic volunteers

MB2 devices were selected for the larger-scale studies because they extracted more blood than MB1 devices, making the composition of the MBs similar to sand fly blood meals. Moreover, samples extracted using MB2 devices yielded 10-fold more DNA than samples extracted with MB1 devices. In the first study MB2s with 0.45 µm pore size PES membranes were employed. However, for the second study, the PES membrane was replaced with Whatman No. 1 filter paper in order to allow blood cells and amastigotes to be absorbed more efficiently. MBs were extracted from two different anatomical sites: face (cheek or zygomatic region) and nape of the neck (Table 2).

**Table 2:** Relationships between microbiopsy type 2 (MB2) samples taken from the face and neck. The first study was in southern Ethiopia and the second study in northern Ethiopia. In both studies, individuals with any level of *Leishmania* parasitemia detected in MBs from the face tended to also have parasitemia in the neck (positive level of agreement – 1st study: *P* = 1·041e-12, Fisher’s Exact Tests (FET) and 2nd study: *P* = 1·257e−06, FET).

	MB Neck +No. (%)	MB Neck −No. (%)
1st study		
MB Face +	58 (32)	27 (14. 9)
MB Face −	16 (8.8)	80 (44.1)

2nd study		
MB Face +	10 (14.5)	7 (10)
MB Face −	3 (4.3)	49 (71)

Asymptomatic volunteers, seropositive for *L*. *donovani* by ELISA, were selected for the first study in southern Ethiopia (*n* = 181, sero-survey conducted 2 months earlier, Hailu et al., unpublished data). Of those, 22 had a history of treatment for VL. A second study was conducted in northern Ethiopia where 69 asymptomatic volunteers were chosen from a roster of individuals who had been screened for VL as part of a cohort study (2010–2015). A majority of the volunteers (54/69) were previously treated for VL. In total, 362 MB2 samples were taken during the first study and 140 during the second. In the first study, 101 individuals were positive by MB2 and 32 were positive by FP. Of these, 22 were positive by both MB2 and FP (Supplementary Fig. S1A, Table 3). In the second study, 20 individuals were positive by MB2 and eight by FP. Only two volunteers were positive by both tests (Supplementary Fig. S1B; Table 3).

**Table 3:** Contingency table showing the association between microbiopsy type 2 (MB2) and finger prick (FP) results. First study (southern Ethiopia); Second study (northern Ethiopia). MB results from the face and neck were merged so that if either yielded a *Leishmania*-positive kinetoplast DNA PCR result, the volunteer was considered positive.

	FP +No. (%)	FP −No. (%)
1st study		
MB +	22 (12.1)	79 (43.6)
MB −	10 (5.5)	70 (38.7)

2nd study		
MB +	2 (2.8)	18 (26)
MB −	6 (8.6)	43 (62.3)

**Fig. 4:**
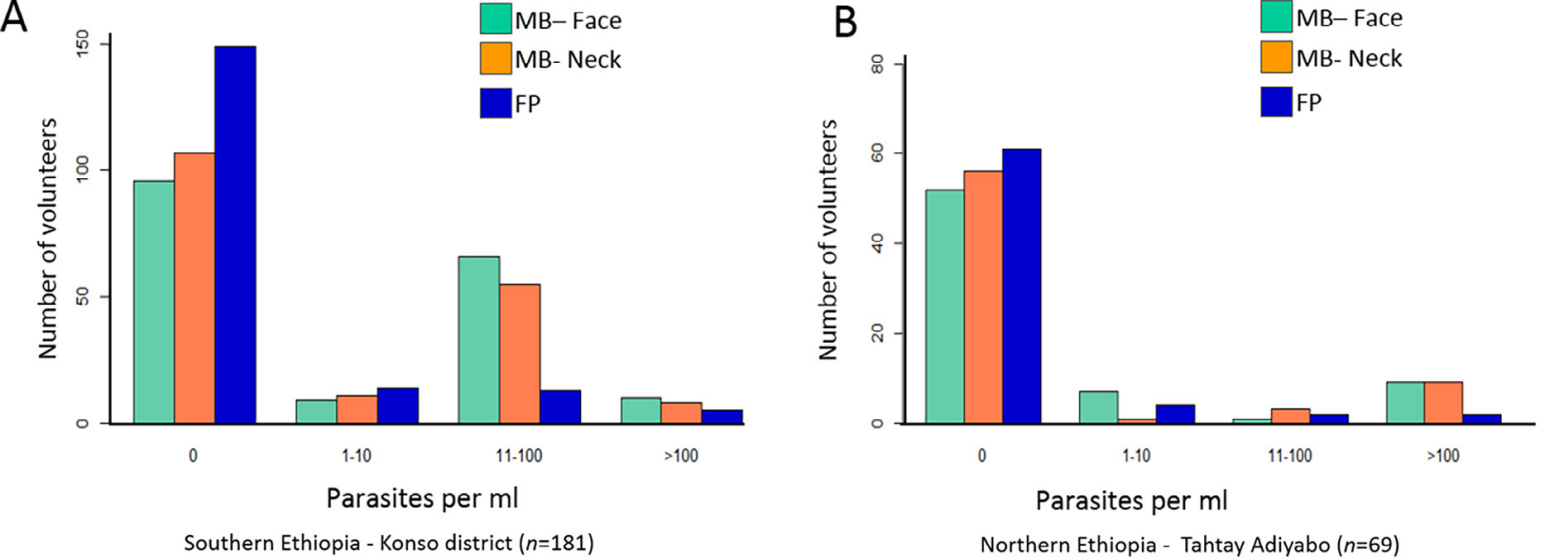
Distribution of parasitemia levels detected by quantitative real time kinetoplast DNA (qRT-kDNA) PCR for *Leishmania donovani* using microbiopsy type 2 (MB2) and finger pricks (FPs). (A) First study (southern Ethiopia) and (B) second study (northern Ethiopia). Calculated parasitemias for MB2s were multiplied by 10 to reflect the small volume of the MBs (∼1.5–3 µl) compared with the filter papers used for the calibration curve (and the FP samples) containing 30–40 µl of blood.

In both studies MB2s detected significantly more infections than FPs (Supplementary Fig. S1A and B), strongly indicating skin, not blood parasitemias, as the source of the *L. donovani* DNA. No agreement between MB2s and FPs was found (first study: *P* = 1·0, 2nd study: *P* = 0.119, FET (Table 3). Positive MB2s were more common on the face than on the neck, although the overall distributions of infection intensities detected by the MB2s did not differ significantly between the two sites (southern Ethiopia study: *P* > 0.582, northern Ethiopia study: *P* = 1·381, FET). In most cases, in both studies, parasitemias were higher in MB2s than FPs (Fig. 4).

In the first study, 58 (32%) individuals were positive by MB2s taken from both the face and the neck, and 80 (44.1%) were negative at both sites. In the second study, 10 volunteers (14·5%) were positive by MB2 at both anatomical sites and 49 (71%) were negative at both sites (Table 2). However, in both studies, MB parasitemias in the face did not significantly correlate with MB parasitemia in the neck (first study: *r^2^* = −0.031, *P* = 0.811; second study: *r^2^* = 0.280, *P* = 0.363).

Interestingly, the odds of testing positive by MB2s were not significantly different for volunteers with histories of VL versus healthy volunteers, and past VL cases were almost as likely as non-VL volunteers to test positive (first study: 95% confidence interval (CI): 0·35–2·59, *P* = 1·0; second study 0·87: 95% CI: 0·22–3·76, *P* = 1). These findings suggest the possible importance of recovered VL patients as reservoirs of *L. donovani*, even when they do not manifest PKDL.

## 4 Discussion

Several studies have shown that *L. donovani* (DNA) is found in the blood of clinical VL cases (Murray et al., 2005). In fact, we found higher parasitemias in FP sampling mostly blood than in MB2 sampling skin and blood, indicating that parasite loads in the blood of clinical VL cases may be higher than those in the skin (Table 1). While transmission from VL patients to sand flies is probably possible, patient numbers are usually low and they are quickly removed from the community for treatment. On the other hand, most infected individuals remain asymptomatic and some of these may serve as reservoir hosts for the anthroponotic transmission of *L. donovani* and possibly for *L. infantum*, although the latter is considered zoonotic (Michel et al., 2011, Miller et al., 2014). The MB devices used in this study were developed in order to determine the reservoir potential (i.e. infectiousness to feeding sand flies) of infected asymptomatic human and animal hosts.

*Leishmania* spp. are not blood parasites per se. Upon their inoculation into the skin by blood-feeding infected sand flies, metacyclic (infective stage) *Leishmania* promastigotes are taken up by resident skin macrophages, dendritic cells and neutrophils where they transform into amastigotes and establish dermal infections (Liu and Uzonna, 2012). Recent experiments in mice and dogs have demonstrated the tendency of *L. donovani* and *L. infantum* infections to maintain long-lasting parasitemias in the skin around the site of the infectious sand fly bite (Pruzinova et al., 2015, Aslan et al., 2016). Moreover, non-ulcerating skin lesions in humans infected with *L. infantum* are also well documented, strongly suggesting that visceralizing *Leishmania* spp. also maintain asymptomatic cutaneous infections at the infective sand fly bite sites (Ponce et al., 1991, Svobodova et al., 2009, Aoun and Bouratbine, 2014). Based on this assumption, we elected to obtain MBs from the face and neck, areas that are always exposed and generally thought to be preferred by biting sand flies, as documented by the frequent incidence of CL lesions on the face, neck and forehead (Williams, 1966, Ponce et al., 1991, Belli et al., 1999, Convit et al., 2005, Svobodova et al., 2009, Aoun and Bouratbine, 2014). Our studies confirmed the frequent presence of dermal (hence accessible to biting sand flies) *L. donovani* infections in residents of endemic regions, regardless of whether they had previous history of VL or not. We postulate that such asymptomatic carriers may be infectious to biting sand fly females.

As noted earlier, the gold standard for determining infectiousness of reservoir hosts to sand fly vectors is xenodiagnosis, which is difficult to implement in the field even if one assumes uniform infectiousness all over the body; not to mention having to feed several batches of sand flies on different parts of the body, including ethically unacceptable sites such as the face. To tackle this problem, we developed the MB devices to replace xenodiagnosis with a minimally invasive and virtually painless surrogate procedure for detecting hosts of *Leishmania* spp. that are potentially infectious to biting sand flies. We elected to use absorbent MB2 devices, since the mixture of blood and lysed skin tissue approximates that of the sand fly blood meal more closely than the piece of skin extracted by MB1 devices (Fig. 1). Initially we used 0.45 µm pore size PES absorbent layers as we observed its dissolution in phenol and presumed this would facilitate efficient DNA extraction. However, for the second study, PES was replaced with inexpensive Whatman No.1 paper that was shown to absorb blood more efficiently and yield more DNA. We are currently developing a rapid, “point of care” molecular diagnostic procedure utilizing loop mediated isothermal amplification (LAMP) to hasten diagnosis and reduce prices (Abbasi et al., 2016).

In a previous study, the reliability of qRT-kDNA PCR for detection of very low parasitemias in repeat DNA extractions from filter paper punches containing 40 µl of blood was >60% (Abbasi et al., 2013). This was explained by the fact that parasite DNA was not dissolved uniformly in blood, and not every paper punch necessarily contained a parasite. In the case of the MB2s, DNA was extracted from the entire volume (∼1·5–3 µl) at once and repeat PCRs were, therefore, always consistent (data not shown). Since all PCR-positive MB samples must have contained at least one “whole” parasite, the effective limit of detection of MBs could not have been lower than one parasite per MB, equivalent to approximately 40–70 parasites/ml. Although hosts with such low concentrations may be poorly infectious to sand flies (Seblova et al., 2013, Miller et al., 2014), sand flies do tend to probe the skin multiple times, thereby increasing the likelihood of infection. In summary, the fact that MBs extracting such small amounts of blood and skin detected infections more frequently than FP blood samples, strongly indicates that skin, not blood, parasitemias were prevalent in the asymptomatic volunteer populations. One remaining question is why there were some (albeit few) volunteers positive by FPs but negative by MB2s. Could there have been skin parasitemias in the fingertips? This seems unlikely since sand flies do not tend to bite the fingertips, but this will need to be investigated further.

To validate the substitution of xenodiagnosis with MBs will require repeated comparisons by feeding insectary-reared sand flies and extracting MBs from multiple locations on the skin of volunteers. Additional parameters such leishmanine skin test (LST) and sero-positivity will also need to be monitored to account for possible naturally acquired transmission-blocking immunity potentially affecting infectiousness (Coutinho-Abreu and Ramalho-Ortigao, 2010). Such a study is already underway on dogs in Brazil, comparing infection rates of *Lutzomyia longipalpis* (vector of *L. infantum*) females that had fed on infected dogs with positivity rates of MBs taken from the same locations (Mota et al., personal communication). Lastly, the usefulness of MBs is not restricted to leishmaniasis. Initial evaluation of MBs for detecting microfilaria of *Onchocerca volvulus* (causative agent of river blindness) showed significant correlation with the accepted diagnostic method using skin snips (study conducted in Ghana by A. Debrah et al., personal communication).
