# Supplementary material for: Minimally invasive microbiopsies: a novel sampling method for identifying asymptomatic, potentially infectious carriers of Leishmania donovani
Source: Int J Parasitol. 2017 Sep;47(10-11):609–16. doi: 10.1016/j.ijpara.2017.02.005 (PMC5596977; doi:10.1016/j.ijpara.2017.02.005)
Supplement: Supplementary data 1 [file mmc1.docx]

Supplementary Figure 1:

Venn Plot depicting the relationships between positive kDNA PCR results obtained using 2 sampling methods, microbiopsy (MB2) and finger prick. **A)** First study (Southern Ethiopia). Most of the positive individual (101/181) were detected using MB2s. Finger pricks were less sensitive at 32/181. 22 individuals were PCR positive by both sampling methods. **B)** Second study (Northern Ethiopia). Here too, MB2s detected more infections (20/69) than FPs (8/69). Only two volunteers were positive by both methods.


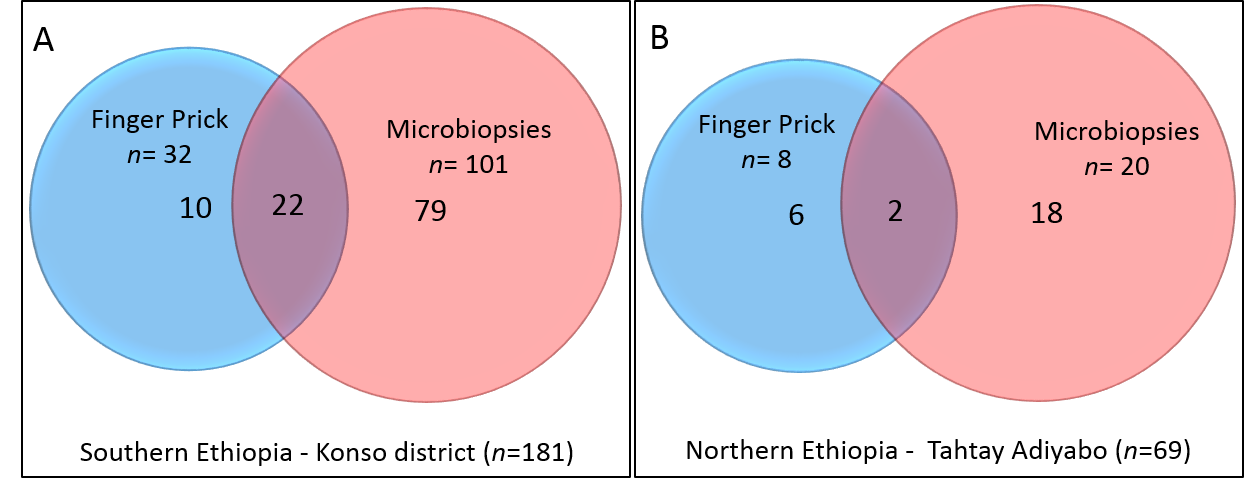


**Supplementary Table 1**

Compilation of the raw data collected during the study

| **Northern Ethiopia - Sehraro** | | | | | | |
| --- | --- | --- | --- | --- | --- | --- |
| Mean number of parasites/ml  in individuals with a past history of VL compared to those without | | | | | | |
|  | MB | FP |  |  |  |  |
| Positive Past VL cases | 177.33 (n=15) | 173.14 (n=7) |  |  |  |  |
| Positive No VL cases | 417 (n=5) | 1 (n=1) |  |  |  |  |
|  |  |  |  |  |  |  |
| Mean number of parasites/ml  in volunteers that were positive in both FP and MB | | | | | | |
|  | MB | FP |  |  |  |  |
| Positive for both (MB & FP) | 37.5 (n=2) | 6 (n=2) |  |  |  |  |
| Positive Past VL | 5 (n=1) | 11 (n=1) |  |  |  |  |
| Positive No VL | 70 (n=1) | 1 (n=1) |  |  |  |  |
|  |  |  |  |  |  |  |
| Mean number of parasites/ml  in volunteers negative by MB but positive by FP or vice versa | | | | | | |
|  | **FP+** | **FP-** |  |  |  |  |
| **MB+** | 37.5 (n=2) | 259.44 (n=18) |  |  |  |  |
| **MB-** | 200.16 (n=6) | 0 (n=43) |  |  |  |  |
|  |  |  |  |  |  |  |
|  |  |  |  |  |  |  |
| **Southern Ethiopia - Aba Roba** | | | | | | |
| Mean number of parasites/ml  in volunteers with a past history of VL compared to those without | | | | | | |
|  | MB | FP |  |  |  |  |
| Positive Past VL | 43.33 (n=12) | 28 (n=1) |  |  |  |  |
| Positive No VL | 77.88 (n=86) | 440.03 (n=31) |  |  |  |  |
|  |  |  |  |  |  |  |
| Mean number of parasites/ml  in volunteers that were positive in both FP and MB | | | | | | |
|  | MB | FP |  |  |  |  |
| Positive for both (MB & FP) | 84.54 (n=22) | 101 (n=22) |  |  |  |  |
| Positive Past VL | 5 (n=1) | 28 (n=1) |  |  |  |  |
| Positive No VL | 88.33 (n=21) | 104.47 (n=21) |  |  |  |  |
|  |  |  |  |  |  |  |
| Mean number of parasites/ml  in volunteers negative by MB but positive by FP or vice versa | | | | | | |
|  | **FP+** | **FP-** |  |  |  |  |
| **MB+** | 84.54 (n=22) | 70.69 (n=79) |  |  |  |  |
| **MB-** | 1144.7 (n=10) | 0 (n=70) |  |  |  |  |
